# Assessing temporal differences of baseline body mass index, waist circumference, and waist-height ratio in predicting future diabetes

**DOI:** 10.3389/fendo.2022.1020253

**Published:** 2023-01-06

**Authors:** Guotai Sheng, Jiajun Qiu, Maobin Kuang, Nan Peng, Guobo Xie, Yuanqin Chen, Shuhua Zhang, Yang Zou

**Affiliations:** ^1^ Jiangxi Provincial Geriatric Hospital, Jiangxi Provincial People’s Hospital, Medical College of Nanchang University, Nanchang, Jiangxi, China; ^2^ Jiangxi Cardiovascular Research Institute, Jiangxi Provincial People’s Hospital, The First Affiliated Hospital of Nanchang Medical College, Nanchang, Jiangxi, China

**Keywords:** waist circumference, BMI, central obesity, diabetes, time-dependent ROC, waist-height ratio

## Abstract

**Objective:**

Obesity is the prominent modifiable risk factor known to influence the occurrence and progression of diabetes other than age, and the objective of this study was to evaluate and compare the predictive value of three simple baseline anthropometric indicators of obesity, body mass index (BMI), waist circumference (WC), and waist-height ratio (WHtR), for the occurrence of diabetes at different time points in the future.

**Methods:**

The study subjects were 12,823 individuals with normoglycemic at baseline who underwent health screening and had measurements of BMI, WC, and WHtR. The outcome of interest was new-onset diabetes during follow-up. Time-dependent receiver operator characteristics (ROC) curves of baseline BMI, WC, and WHtR for predicting the risk of diabetes in the next 2 to 12 years were constructed and their area under the ROC curves (AUCs) and corresponding optimal threshold values were calculated for each time point, which were used to compare the accuracy and stability of the above three indicators for predicting the occurrence of diabetes in different future periods.

**Results:**

During a median follow-up period of 7.02 years, with a maximum follow-up of 13 years, 320 new-onset diabetes were recorded. After adjusting for confounders and comparing standardized hazard ratios (HRs), WC was shown to be the best simple anthropometric indicator of obesity reflecting diabetes risk in all models, followed by WHtR. Time-dependent ROC analysis showed that WC had the highest AUC in predicting the occurrence of diabetes in the short term (2-5 years), and WHtR had the highest AUC in predicting the occurrence of diabetes in the medium to long term (6-12 years), while in any time point, both WC and WHtR had higher AUC than BMI in predicting future diabetes. In addition, we found relatively larger fluctuations in the thresholds of BMI and WC for predicting diabetes over time, while the thresholds of WHtR consistently remained between 0.47-0.50; comparatively speaking, WHtR may have greater application value in predicting future diabetes.

**Conclusions:**

Our analysis sustained that central obesity is a more important predictor of diabetes, and in clinical practice, we proposed measuring WHtR as a useful tool for predicting future diabetes.

## Introduction

Diabetes, one of the leading causes of death and disability, is now very prevalent worldwide, generating an economic burden of approximately 10% of global health expenditures (1, 2). There are many risk factors contributing to the development of diabetes, the most common of which are obesity, advanced age, family history of diabetes, race, lipid abnormalities, poor eating habits and lack of physical activity (3, 4). It is worth noting that age, race, and family history of diabetes cannot be changed, while obesity is generally considered to be an adverse consequence of poor eating habits and insufficient physical exercise, and dyslipidemia is a common metabolic abnormality in obese people (5, 6). Therefore, it is very important to effectively evaluate the relationship between obesity and diabetes to reduce the risk of diabetes.

BMI, WC, and WHtR are the simplest general anthropometric indicators of obesity used to assess diabetes risk (7–9), where BMI is calculated as weight(kg)/[height(m)]^2^ and WHtR is calculated as WC(cm)/height(cm). Compared with BMI, evidence from recent observational studies has shown that measures of central obesity, WC and WHtR, are more strongly associated with diabetes and its associated cardiovascular disease risk because they take into account the distribution of fat (10–15). Furthermore, it is worth noting that in a recent meta-analysis of 216 cohort studies published in the BMJ, Jayedi et al. showed that in routine measurements, WHtR was more associated with diabetes than WC, waist-to-hip ratio and BMI (16). Although the correlation between simple obesity parameters and diabetes has been well unified, among which WHtR was considered to be the most appropriate index, there is no unified answer to the prediction of future diabetes by simple obesity parameters at present, and the viewpoints in several published meta-analyses were also inconsistent or even contradictory (9, 16–22). It is also important to note that although most of the published similar studies performed follow-up, these studies did not factor time into the ROC analysis (9, 18, 21), which may have some bias on the predictive results of the longitudinal analysis. To address this issue, in the current study, we constructed time-dependent ROC curves at multiple follow-up time points, based on a large longitudinal cohort of NAGALA, to evaluate the variations in predictive values of BMI, WC, and WHtR for future diabetes.

## Methods

### Study population and data sources

**Figure 1:**
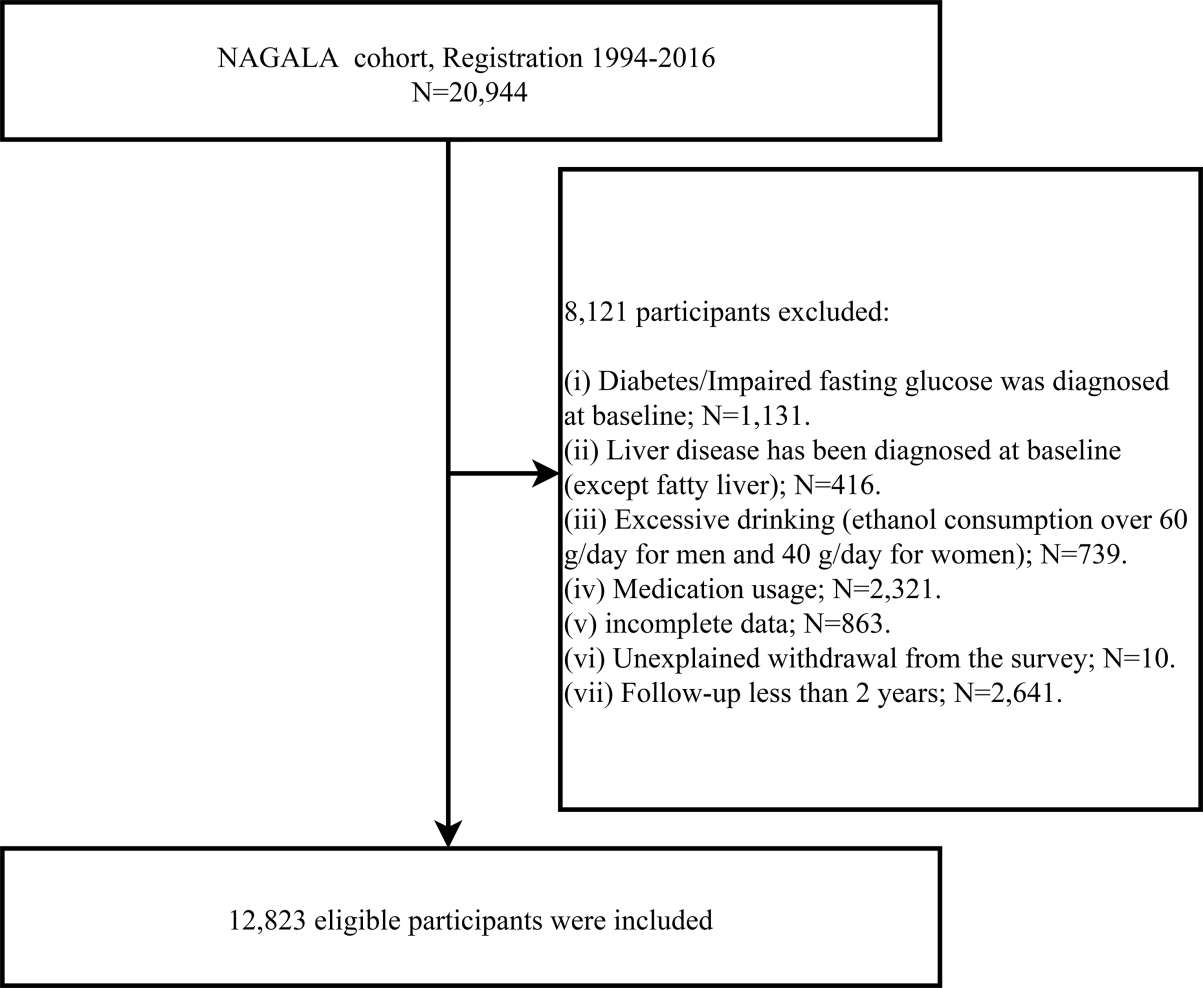
Study profile.

The population for this study was drawn from a longitudinal cohort study, the NAGALA study, in which we used data from 1994-2016 (Containing 20,944 participants) for an investigation into the predictive power of simple anthropometric indicators of obesity for future diabetes risk. This longitudinal cohort data has been uploaded to the Dryad database for public sharing by Okamura et al. (23), and according to the terms of service of the Dryad database, researchers can employ these data for secondary analysis based on different study hypotheses. In a previous study, Okamura et al. analyzed the association between obesity phenotype and diabetes, and the detailed study design has been published elsewhere (24). In short, the NAGALA cohort, initiated in 1994, continues to enroll the general population who have participated in a health check-up program at the Murakami Memorial Hospital Check-up Center to conduct an epidemiological study of diabetes and fatty liver disease. It should be mentioned that about 60% of the participants in the NAGALA cohort received one or two health check-ups per year, and the vast majority of participants underwent repeated check-ups (including blood glucose as well as abdominal ultrasound) at follow-up. During each physical examination, the participants were reviewed by trained investigators for diabetes diagnosis, including whether the participants had diabetes diagnosed by other medical staff during the follow-up period, and blood glucose parameters were assessed. In this study, based on the new research hypothesis, we selected the target population of the current study according to the following exclusion criteria: (a) subjects with diabetes/impaired fasting glucose (6.1 mmol/L< baseline FPG <7.8 mmol/L)/liver disease (except fatty liver) at baseline; (b) subjects with alcohol abuse (>60 g per day for men and >40 g per day for women) (25); (c) subjects who were taking medication at baseline; (d) subjects with incomplete physical examination data; (e) subjects who withdrew from the survey for unknown reasons. To minimize the potential effect of reverse causality, we also excluded subjects with less than 2 years of follow-up data (26). Finally, we included 12,823 eligible subjects, and **Figure 1** shows the process of inclusion and exclusion of the study population.

### Ethical approval and consent to participate

The previous study was approved by the Ethics Committee of Murakami Memorial Hospital and authorized with informed consent from the subjects (24). In the present study, as it was a *post hoc* analysis of the NAGALA study, the Ethics Committee of Jiangxi Provincial People’s Hospital waived repeated acquisition of informed consent from the subjects and approved the current study protocol (IRB2021-066), and all study procedures were performed in accordance with the Declaration of Helsinki. STROBE checklist (S1Text).

### Data acquisition and measurement

As reported in the previous study (24), for demographic information, subjects reported factors such as gender, age, drinking/smoking status, exercise habits, medication use, and disease status through a standardized questionnaire. Anthropometric indicators including height, weight, WC, and blood pressure were measured by trained professionals using standard methods.

Venous blood samples were collected from the subjects after 8 hours of fasting by experienced medical personnel, and then biochemical markers such as liver enzymes [gamma-glutamyl transferase (GGT), aspartate aminotransferase (AST), alanine aminotransferase (ALT)], lipid indicators [high-density lipoprotein cholesterol (HDL-C), triglycerides (TG), total cholesterol (TC)], and blood glucose indicators [hemoglobin A1c (HbA1c), fasting plasma glucose (FPG)] were measured using an automated biochemical analyzer.

Fatty liver was determined by experienced gastroenterologists based on several sonograms of deep attenuation, hepatorenal echo contrast, vascular blurring, and liver brightness under abdominal ultrasound (27).

### Definition

Exercise habit: Having more than 1 regular exercise session per week was defined as having an exercise habit, otherwise, it was considered as no exercise habit (28).

Drinking status: Professionals assessed alcohol intake by asking subjects about their weekly drinking in the month prior to the survey and classified subjects into four categories based on the following criteria: <40 g/week, no or little drinking; 40-140 g/week, light drinking; 140-280 g/week, moderate drinking; >280 g/week, heavy drinking (25).

Smoking status: Smokers were divided into three groups: non-smokers, past smokers, and current smokers based on the participants’ smoking history before the baseline survey.

Diabetes determination: According to the American Diabetes Association criteria (29), subjects were defined as having diabetes if they tested FPG ≥ 7.0 mmol/L or HbA1c ≥ 6.5% during follow-up or if they were diagnosed with diabetes by other medical personnel.

### Statistical analysis

The current research data were analyzed using R language version 4.2.0. The median, mean, or proportions of demographic and biochemical factors in the study population were summarized by grouping according to the presence or absence of diabetes during follow-up. Using the inverse probability of treatment weighting method to calculate the weighted standardized difference to estimate the magnitude of the difference between groups at baseline (the difference > 10%) was considered significant) (30, 31).

Cox proportional hazards regression models were used to assess the associations between baseline BMI, WC, WHtR and the risk of developing diabetes. Before modeling for data analysis, the possibility of multicollinearity between independent variables and covariates was tested using a variance inflation factor, where a variance inflation factor of less than 5 was considered desirable (32). In addition, the proportional hazards assumptions were assessed using Kaplan-Meier survival curves (33). Five multivariate Cox regression models were developed based on the epidemiological STROBE statement (34). The effects of age and gender on outcomes were considered in model 1. Model 2 was further adjusted for height and lifestyle-related factors such as exercise habits, smoking status, drinking status, and fatty liver. On this basis, model 3 additionally adjusted for liver enzyme parameters (ALT, AST, GGT). Model 4 adjusted all non-collinear covariates that were potentially associated with diabetes. Finally, in order to further consider the potential effects of insulin resistance (IR), we calculated the triglyceride-glucose index (35), an alternative indicator of IR, and adjusted this variable in model 5. Furthermore, we also used R-packet timeROC to construct ROC curves at 11 follow-up time points; then to calculate the AUC for each parameter from year 2 to year 12 and record the corresponding optimal thresholds for comparing the predictive ability and stability of BMI, WC and WHtR for predicting diabetes in different future periods, and finally compared the AUC of BMI, WC, WHtR at each time point. A 2-tailed value of *P*<0.05 was considered significant.

## Results

### Baseline characteristics of the study population

Data from 12,823 subjects were finally analyzed according to exclusion criteria, with a mean age of 43.54 (8.70) years. During a median follow-up period of 7.02 years (maximum 13 years), new-onset diabetes was recorded in 320 participants, with an incidence density of 34.93 per 10,000 person-years. We divided the population into diabetic and non-diabetic groups based on whether the subject developed diabetes during follow-up and descriptively compared the differences in clinical baseline characteristics between the two groups. As shown in **Table 1**, significant differences between those with and without future diabetes were already present at the time of the initial collection of baseline information (all standardized differences were >10%). Compared with the non-diabetic group, subjects in the diabetic group were older, more men, more alcohol consumption, and more of them had a history of smoking (including past and current smoking) and also a higher prevalence of fatty liver at baseline (105%). In terms of baseline glucose and lipid metabolism-related parameters, FPG, HbA1c, TG, and TC were significantly higher in the diabetic group than in the non-diabetic group; in terms of anthropometric parameters, weight, WC, BMI, and WHtR also differed significantly between the two groups (79%-96%).

**Table 1:** Baseline demographic, lifestyle, and laboratory characteristics in subjects with and without diabetes.

	Non-diabetic	Diabetic	Standardized Difference (95% CI), %
Participants(n)	12503	320	
Age(years)	43.46 (8.69)	46.83 (8.38)	39 (28, 51)
Gender			48 (37, 59)
Women	5728 (45.81%)	75 (23.44%)	
Men	6775 (54.19%)	245 (76.56%)	
Height (m)	1.65 (0.08)	1.66 (0.09)	17 (5, 28)
Weight (kg)	60.38 (11.44)	70.24 (13.58)	79 (67, 90)
BMI (kg/m^2^)	22.03 (3.05)	25.23 (3.90)	91 (80, 103)
WC (cm)	76.16 (8.98)	85.45 (10.38)	96 (85, 107)
WHtR	0.46 (0.05)	0.51 (0.06)	96 (85, 107)
ALT (IU/L)	17.00 (13.00-23.00)	25.00 (19.00-39.25)	69 (58, 80)
AST (IU/L)	17.00 (14.00-21.00)	20.00 (16.00-26.00)	42 (31, 53)
GGT (IU/L)	15.00 (11.00-22.00)	24.00 (17.00-36.00)	49 (38, 60)
HDL-C (mmol/L)	1.45 (0.39)	1.18 (0.31)	79 (67, 90)
TC (mmol/L)	5.11 (0.85)	5.44 (0.87)	38 (27, 49)
TG (mmol/L)	0.73 (0.50-1.11)	1.24 (0.88-1.96)	75 (64, 86)
HbA1c (%)	5.15 (0.32)	5.49 (0.36)	102 (91, 113)
FPG (mmol/L)	5.14 (0.41)	5.60 (0.36)	122 (110, 133)
SBP (mmHg)	114.24 (14.81)	122.45 (15.82)	54 (43, 65)
DBP (mmHg)	71.48 (10.33)	77.47 (10.23)	58 (47, 69)
Fatty liver	2036 (16.28%)	197 (61.56%)	105 (94, 116)
Exercise habits	2153 (17.22%)	41 (12.81%)	12 (1, 23)
Drinking status			20 (9, 31)
No/little	9581 (76.63%)	230 (71.88%)	
Light	1432 (11.45%)	34 (10.62%)	
Moderate	1075 (8.60%)	32 (10.00%)	
Heavy	415 (3.32%)	24 (7.50%)	
Smoking status			47 (36, 58)
Non	7378 (59.01%)	121 (37.81%)	
Past	2345 (18.76%)	66 (20.62%)	
Current	2780 (22.23%)	133 (41.56%)	

Values were expressed as mean (SD) or medians (quartile interval) or n (%). BMI, body mass index; WC, Waist circumference; WHtR, Waist-to-height ratio; ALT, alanine aminotransferase; AST, aspartate aminotransferase; GGT, gamma-glutamyl transferase; HDL-C, high-density lipoprotein cholesterol; TC, total cholesterol; TG, triglyceride; HbA1c, hemoglobin A1c; FPG, fasting plasma glucose; SBP, systolic blood pressure; DBP, diastolic blood pressure.

### Associations between BMI, WC, and WHtR and new-onset diabetes

Before building the Cox proportional hazards regression models, our data did not violate the proportional hazards assumptions based on the Kaplan-Meier graphical method for BMI quartiles, WC quartiles, and WHtR quartiles over time [see **Additional File 1**, **Supplementary Figures 1-3**]; moreover, covariates that were collinear with the independent variables will not be included in the subsequent multivariate Cox regression models [see **Additional File 1**, **Supplementary Tables S1-S3**].

Multivariate Cox regression models were developed to assess the associations between baseline BMI, WC, and WHtR and the occurrence of future diabetes. To standardize the hazard ratio (HR) of each independent variable affecting the outcome, we performed Z-transformation for BMI, WC, and WHtR before incorporating them into the Cox regression models. Based on epidemiology, we established five multivariate Cox regression models (**Table 2**), from model 1 to model 5 showing the dynamic changes in HR after stepwise adjustment. From the overall trend, the HR values of the three simple obesity indicators decreased gradually with the increase of covariates, and compared with WHtR and BMI, WC reflected a higher risk of developing diabetes in the future. In the final model (model 5), the risk of diabetes increased by 39% [95% confidence interval (CI): 1.23-1.56] for each standard deviation (SD) increase in BMI, 52% (95% CI: 1.33-1.75) for each SD increase in WC, and 47% (95% CI: 1.29-1.66) for each SD increase in WHtR.

**Table 2:** Cox regression analyses for the association between BMI, WC, WHtR and incident diabetes in different models.

	Hazard ratios (95% confidence interval)				
	Model 1	Model 2	Model 3	Model 4	Model 5
BMI (Per SD increase)	2.15 (1.98, 2.33)	1.67 (1.51, 1.85)	1.61 (1.45, 1.79)	1.38 (1.22, 1.55)	1.39 (1.23, 1.56)
WC (Per SD increase)	2.37 (2.15, 2.62)	1.85 (1.64, 2.09)	1.78 (1.57, 2.01)	1.51 (1.32, 1.73)	1.52 (1.33, 1.75)
WHtR (Per SD increase)	2.32 (2.12, 2.54)	1.76 (1.58, 1.96)	1.69 (1.51, 1.89)	1.46 (1.29, 1.65)	1.47 (1.29, 1.66)

BMI, body mass index; WC, waist circumference; WHtR, waist-to-height ratio.

Model 1 adjusted for gender, age.

Model 2 adjusted for gender, age, height, fatty liver, exercise habits, drinking status and smoking status.

Model 3 adjusted for gender, age, height, fatty liver, exercise habits, drinking status. smoking status, ALT, AST and GGT.

Model 4 adjusted for gender, age, height, fatty liver, exercise habits, drinking status, smoking status, ALT, AST, GGT, HDL-C, TC, TG, FPG, HbA1c and SBP.

### Accuracy of BMI, WC, and WHtR in predicting the occurrence of diabetes in different future periods

Time-dependent ROC curves were plotted for assessing the accuracy of baseline BMI, WC, and WHtR in predicting the onset of diabetes at different times in the future, and the corresponding AUCs were shown in **Table 3**. Generally speaking, the simple obesity indicators, BMI, WC, and WHtR, were all highly stable in predicting future diabetes, and interestingly, the predictive accuracy of WHtR increased progressively with a longer follow-up time. It is worth mentioning that the AUC of WC was higher than that of BMI and WHtR in predicting the occurrence of diabetes in the year 2 to year 5 of follow-up [AUC:(2-years: WC 0.67 > WHtR 0.64 > BMI 0.63; 3-years: WC 0.70 > WHtR 0.68 > BMI 0.67; 4-years: WC 0.67 > WHtR 0.66 > BMI 0.64; 5-years: WC 0.70 > WHtR 0.69 > BMI 0.66)], and the AUC of WHtR was the highest in predicting the occurrence of diabetes in the year 6 to year 12 of follow-up [AUC:(6-years: WHtR 0.70 = WC 0.70 > BMI 0.68; 7-years: WHtR 0.72 = WC 0.72 > BMI 0.69; 8-years: WHtR 0.72 > WC 0.71 > BMI 0.70; 9-years: WHtR 0.71 > WC 0.70 > BMI 0.69; 10-years: WHtR 0.71 > WC 0.69 = BMI 0.69; 11-years: WHtR 0.71 > WC 0.69 = BMI 0.69; 12-years: WHtR 0.70 > WC 0.67 = BMI 0.67)]. In addition, the pairwise comparison results based on different time points showed that BMI and WC differed significantly only at the second- and fifth-year time points, BMI and WHtR differed from year 7 to year 12, and WC and WHtR only differed at year 11 and year 12. Although most of the results of the statistical pairwise comparison showed that these differences were not significant, it is undeniable that at all time points, the AUC of WC and WHtR for predicting future diabetes was higher than that of BMI.

**Table 3:** Best threshold, sensitivities, specificities and areas under the time-dependent receiver operating characteristic curves for BMI, WC and WHtR predicting future diabetes risk.

		BMI				WC				WHtR			
	Diabetes events, n	Optimal threshold	AUC	Sensitivity	Specificity	Optimal threshold	AUC	Sensitivity	Specificity	Optimal threshold	AUC	Sensitivity	Specificity
2-years	38	20.99	0.63*	0.85	0.39	79.5	0.67	0.63	0.64	0.48	0.64	0.56	0.68
3-years	29	24.57	0.67	0.44	0.81	82.1	0.70	0.76	0.75	0.48	0.68	0.63	0.68
4-years	34	23.89	0.64	0.45	0.75	82.1	0.67	0.50	0.75	0.48	0.66	0.63	0.63
5-years	31	23.51	0.66*	0.54	0.71	83.6	0.70	0.54	0.79	0.50	0.69	0.54	0.76
6-years	39	23.41	0.68	0.57	0.70	79.7	0.70	0.65	0.65	0.48	0.70	0.63	0.68
7-years	32	22.26	0.69	0.74	0.57	80.1	0.72	0.66	0.68	0.49	0.72**	0.58	0.75
8-years	20	22.01	0.70	0.78	0.53	80.5	0.71	0.63	0.69	0.49	0.72**	0.63	0.71
9-years	34	22.00	0.69	0.76	0.53	79.3	0.70	0.67	0.64	0.49	0.71**	0.57	0.73
10-years	28	22.03	0.69	0.75	0.54	79.5	0.69	0.65	0.65	0.47	0.71**	0.73	0.59
11-years	22	22.00	0.69	0.75	0.54	79.6	0.69***	0.64	0.66	0.47	0.71**	0.72	0.60
12-years	13	21.11	0.67	0.84	0.42	79.7	0.67***	0.61	0.66	0.47	0.70**	0.68	0.62

AUC, area under the curve; other abbreviations as in **Table 1**.

*P < 0.05 BMI vs WC; **P < 0.05 BMI vs WHtR; ***P < 0.05 WC vs WHtR; Other variables with no special mark on the upper right corner had P values > 0.05 after pair-wise comparison.

### Threshold analysis of BMI, WC, and WHtR for predicting the onset of diabetes in different future periods

**Figure 2:**
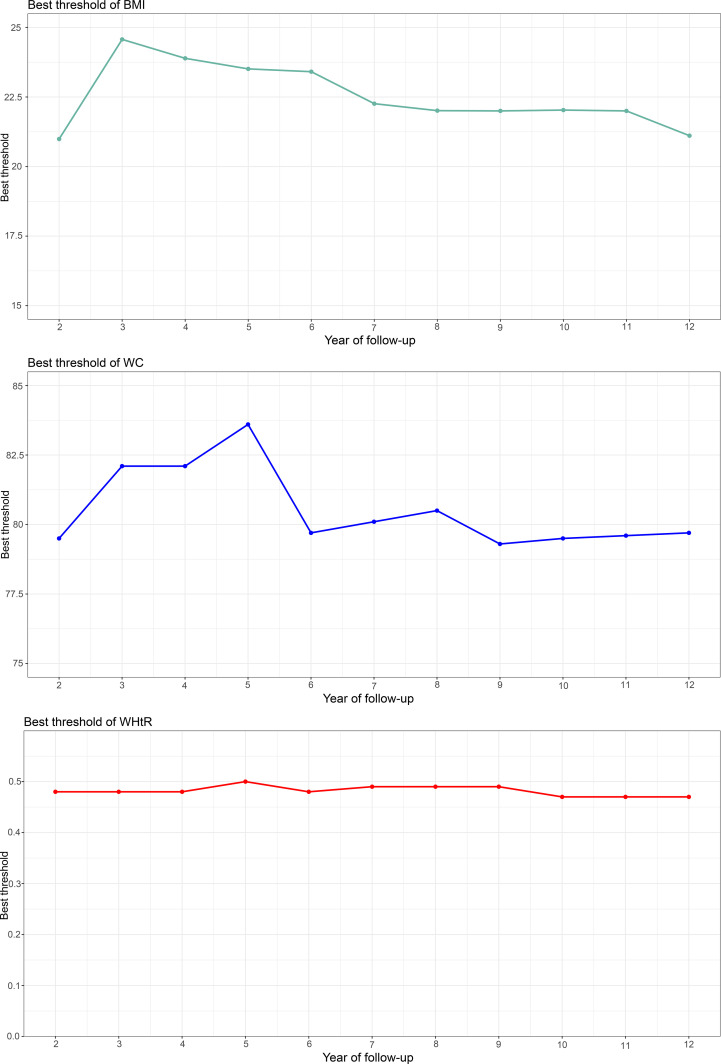
Threshold fluctuation of BMI, WC and WHtR used to predict future diabetes risk. BMI, body mass index; WC, waist circumference; WHtR, waist-height ratio.

Using time-dependent ROC curves, we also calculated the optimal thresholds and the corresponding sensitivities and specificities of baseline BMI, WC, and WHtR for predicting diabetes at different times in the future (**Figure 2**). We found large fluctuations in the optimal thresholds of BMI and WC for predicting diabetes in different future periods (BMI optimal thresholds range: 20.99-24.57; WC optimal thresholds range: 79.3-83.6), while the thresholds of WHtR remained relatively stable (WHtR optimal thresholds range: 0.47-0.5). In addition, it is worth mentioning that the sensitivity of BMI for predicting future diabetes gradually increased and the specificity gradually decreased over time; while the sensitivity and specificity of WC, and WHtR for predicting future diabetes fluctuated relatively little at multiple time points.

## Discussion

This longitudinal cohort study explored the relationship between simple baseline anthropometric indicators of obesity, BMI, WC, and WHtR, and the risk of developing diabetes in different future periods. The study found that WC and WHtR may have better application value than BMI in assessing the risk of diabetes and predicting future diabetes.

The correlation between simple measurement of obesity parameters and diabetes has been widely studied before. WC and WHtR, which represent central obesity, have been recognized by most researchers as better indicators than BMI (10–16). In the current study, we employed a similar approach to that adopted in several previous meta-analyses, and our findings also supported a more important role of central obesity in the development of diabetes, where baseline WC may be the most useful simple obesity indicator to assess the risk of future diabetes, a finding that was consistent with the results of Prof. Kodama’s as well as Prof. Hartwig’s meta-analysis (19, 21). These findings collectively suggested that screening for central obesity should be given greater attention in diabetes risk assessment.

The diagnostic/predictive value of BMI, WC, and WHtR for diabetes had been summarized in several previous meta-analyses. Generally speaking, WHtR and WC were equivalent and both were superior to BMI (9, 18, 21); further distinguishing meta-analyses of cohort studies showed that WHtR was the best predictor (21). It is important to note that although most of the studies included in several published meta-analyses have performed follow-up, they did not factor time into the ROC analysis in assessing the predictive power of simple anthropometric indicators of obesity for diabetes (9, 18, 21), and only one study specially assessed the accuracy of these simple indicators for predicting the occurrence of diabetes at 5 years (22). In a meta-analysis of 21 cohort studies by Lee et al., they used Harrell’s-index to test the predictive accuracy of simple obesity parameters for the risk of diabetes; and the results showed that there was no significant difference between WHtR, WC, and BMI in terms of the predictive accuracy of 5-year diabetes (22). In the current study, we used time-dependent ROC curves, in a cohort of 12,823 nondiabetic subjects, to evaluate the accuracy of baseline BMI, WC, and WHtR in predicting new-onset diabetes at different time points in the future, and the results showed that in terms of predicting diabetes at five years, both WC and WHtR had AUCs greater than BMI, which was different from the conclusion of Lee et al. Based on the results of the above analysis, we further analyzed the accuracy of using baseline BMI, WC, and WHtR for predicting diabetes at each time point from year 2 to year 12, and the results showed that WC had the highest AUC in predicting the onset of diabetes in the short term (2-5 years), and WHtR had the highest AUC in predicting the onset of diabetes in the medium and long term (6-12 years), while in any time point, both WC and WHtR had higher AUC than BMI in predicting future diabetes. In general, an early assessment of central obesity status may be more beneficial for the primary prevention of diabetes than an assessment of general obesity.

We further evaluated the predictive thresholds of these simple obesity parameters for the occurrence of diabetes at each time point in the next 2-12 years. The results showed that in the same population the threshold of WHtR for predicting future diabetes was more stable over time than BMI/WC and may have better application value. Threshold analyses regarding using simple anthropometric indicators of obesity for the diagnosis/prediction of diabetes have also been reported in several previous studies (18, 20, 36–39), and in general, there were large differences in BMI and WC thresholds among different ethnic groups, while the threshold of WHtR was relatively stable. However, it is worth noting that according to the results of the meta-analysis by Savva et al. the median threshold of WHtR for identifying/predicting diabetes in Asian populations was 0.51 (20), a result similar to that of the current study by time-dependent ROC analysis; moreover, in the study by Savva et al., they further distinguished a WHtR threshold of 0.56 for identifying/predicting diabetes in non-Asian populations. These findings suggested that there may be small racial differences in WHtR for diagnosing/predicting diabetes risk, and further evaluation of the threshold of WHtR for predicting diabetes using time-dependent ROC in other races is needed to verify the stability of this finding.

As a chronic disease, early detection of potential risk factors and maintaining a healthy lifestyle are key to preventing diabetes (40), and screening for diabetes using non-invasive and simple anthropometric indicators is of greater importance compared to the drawing of venous blood for glucose measurement. Our results in the current study supported the idea that central obesity can provide additional information in terms of diabetes risk. In clinical practice, we suggested that more attention needs to be paid to those simple obesity indicators WC and WHtR which represent central obesity and have the most direct relevance for diabetes prevention. Furthermore, considering the performance of these simple obesity indicators for predicting diabetes at different time points, that is, the stability of predictive values and threshold fluctuations, we believed that WHtR was the best predictive indicator for diabetes. Our findings supported the public health initiative ‘to keep WC at less than half of your height’ (41) and demonstrated that this initiative is applicable at any time point. Referring to Professor Ashwell’s vivid description, we only need a rope in the prediction of diabetes: use the rope to mark the height, fold it in half, and then wrap it around the waist. Also, according to the available literature, we have learned that WHtR is not only a good surrogate indicator of visceral fat compared to WC, but also has a great practical advantage in eliminating differences in body size among different ethnic groups, and using 0.5 as a threshold is generally suitable for health risk screening of the whole population, including children and adults (18, 42).

The main limitations of this study are found in the following (1): Waist-to-hip ratio is also useful in the assessment of central obesity among the simple anthropometric indicators of obesity (43, 44), however, hip circumference was not assessed in the current study, so it was not possible to further compare the predictive power of waist-to-hip ratio with BMI, WC, and WHtR for future diabetes. It is worth mentioning that several published meta-analyses generally support the superiority of WHtR over waist-to-hip ratio both in assessing diabetes risk and in identifying/predicting diabetes (16, 19, 21, 22). In addition, the influence of the history of gestational diabetes has not been taken into account in the current study, which may have an impact on the results of some women (2). The current study was a single-center cohort study, so the applicability of the findings to other races needs to be further validated. From another perspective, however, the single-center cohort ensured a good homogeneity of the study population (45) and yielded relatively reliable findings. Combined with the results of the meta-analysis by Savva et al. and the current analysis (20), the findings of the current study were applicable at least in Asian populations (3). The study population in the current study did not measure 2-hour postprandial glucose, therefore, we may have missed some of the diabetic patients (4). The survival status of the subjects was not recorded in the current study, thus some death cases during the follow-up period may pose some competing risks to the current study results (5). Because repeated-measures data of baseline indicators in the follow-up period were not included in the current study, the impact of dynamic changes in simple measures of obesity on diabetes cannot be further assessed, and further research is needed.

## Conclusion

Altogether, our findings confirmed that central obesity was a more important risk/predictive factor for the assessment of diabetes than general obesity and identified WHtR as the most practical simple anthropometric indicator of obesity for predicting future diabetes, with 0.5 serving as a threshold value for initial diabetes risk screening.

## Data availability statement

The original contributions presented in the study are included in the article/**Supplementary Material**, further inquiries can be directed to the corresponding author.

## Ethics statement

The studies involving human participants were reviewed and approved by The Ethics Committee of Jiangxi Provincial People’s Hospital. Written informed consent for participation was not required for this study in accordance with the national legislation and the institutional requirements.

## Author contributions

All authors conceived and designed the study. YZ, MK, GS, SZ and JQ conducted statistical analyses, and all authors interpreted the findings. YZ, GS, MK and JQ drafted the manuscript. GX, SZ, YC and NP critically reviewed the manuscript for key intellectual content. All authors approved the final manuscript. YZ and GS are the guarantors and, as such, had full access to the data and take responsibility for its integrity and accuracy. All authors contributed to the article and approved the submitted version.
