# Supplementary material for: Assessing temporal differences of baseline body mass index, waist circumference, and waist-height ratio in predicting future diabetes
Source: Front Endocrinol (Lausanne). 2023 Jan 6;13:1020253. doi: 10.3389/fendo.2022.1020253 (PMC9852880; doi:10.3389/fendo.2022.1020253)
Supplement: Supplementary file 1 [file DataSheet_1.docx]

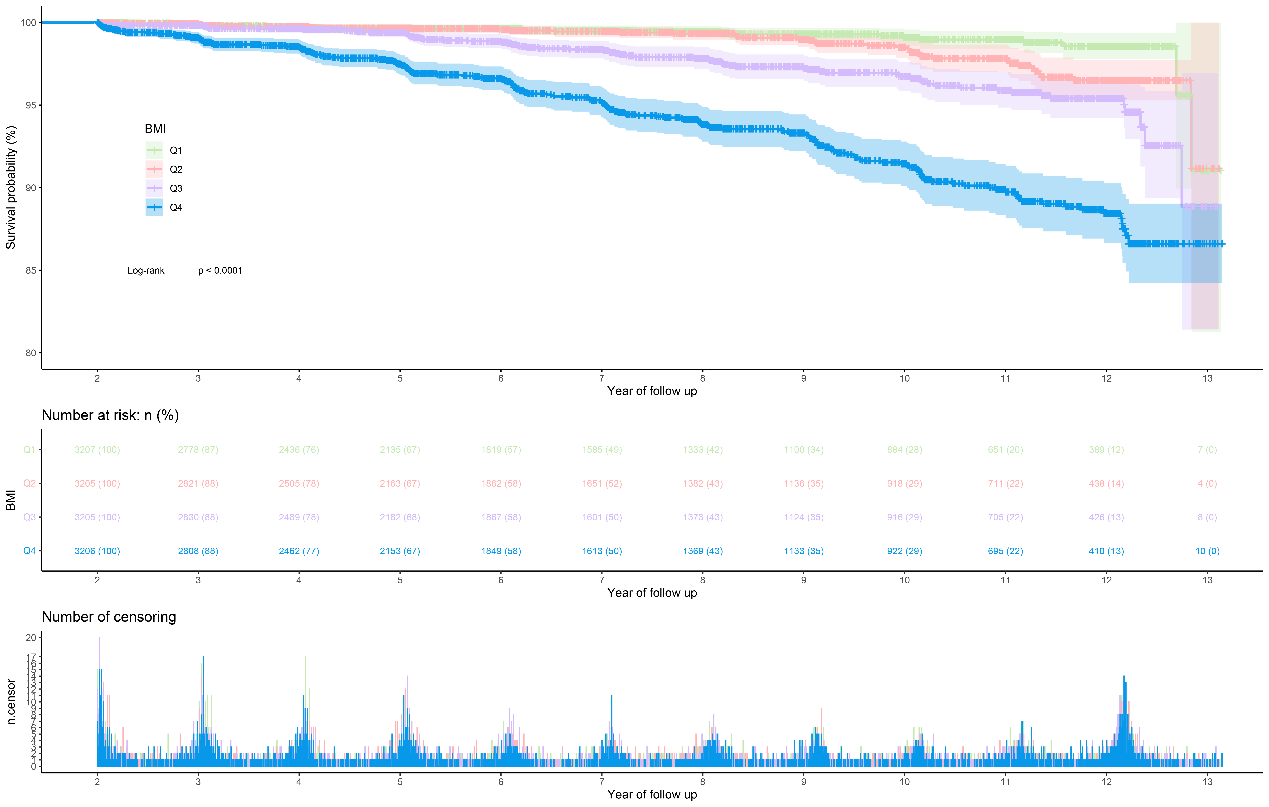


**Figure S1:** Kaplan-meier curve of BMI quartiles over time. BMI: Body mass index.


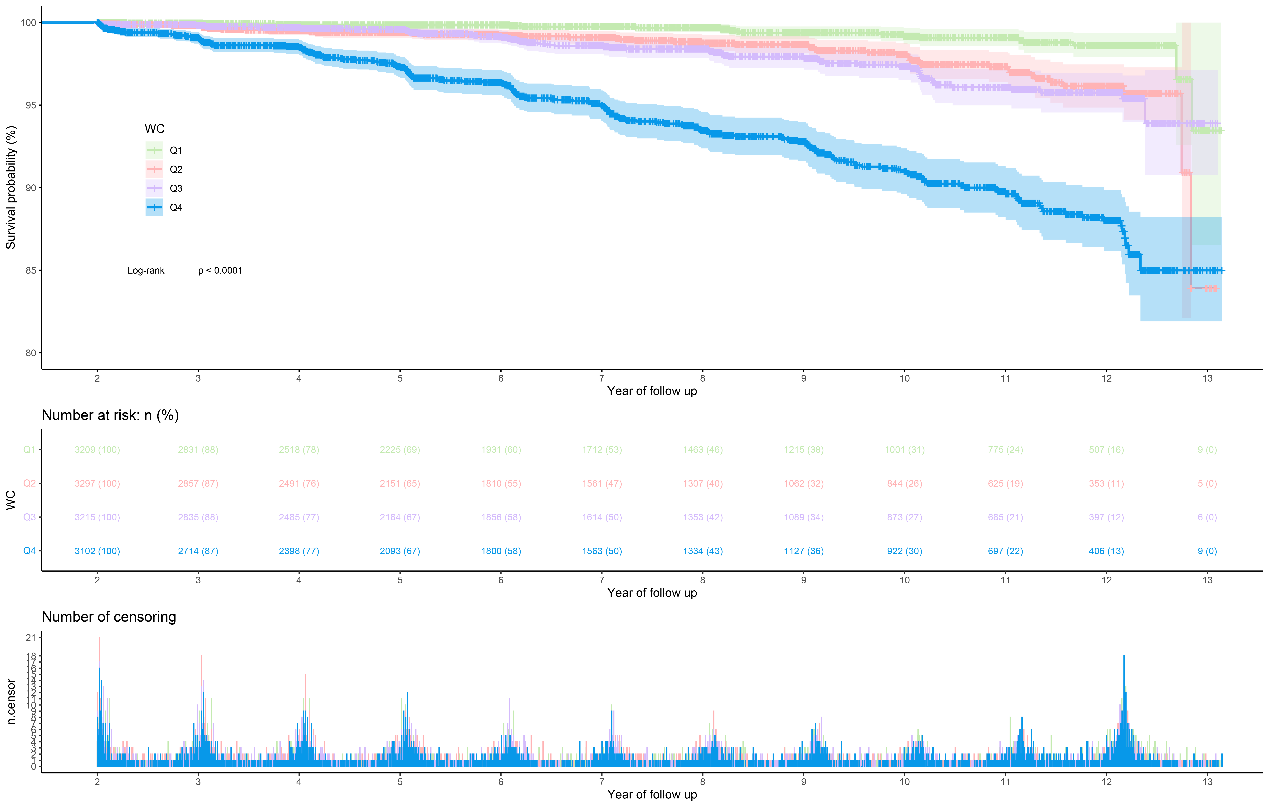


**Figure S2:** Kaplan-meier curve of WC quartiles over time. WC: waist circumference;


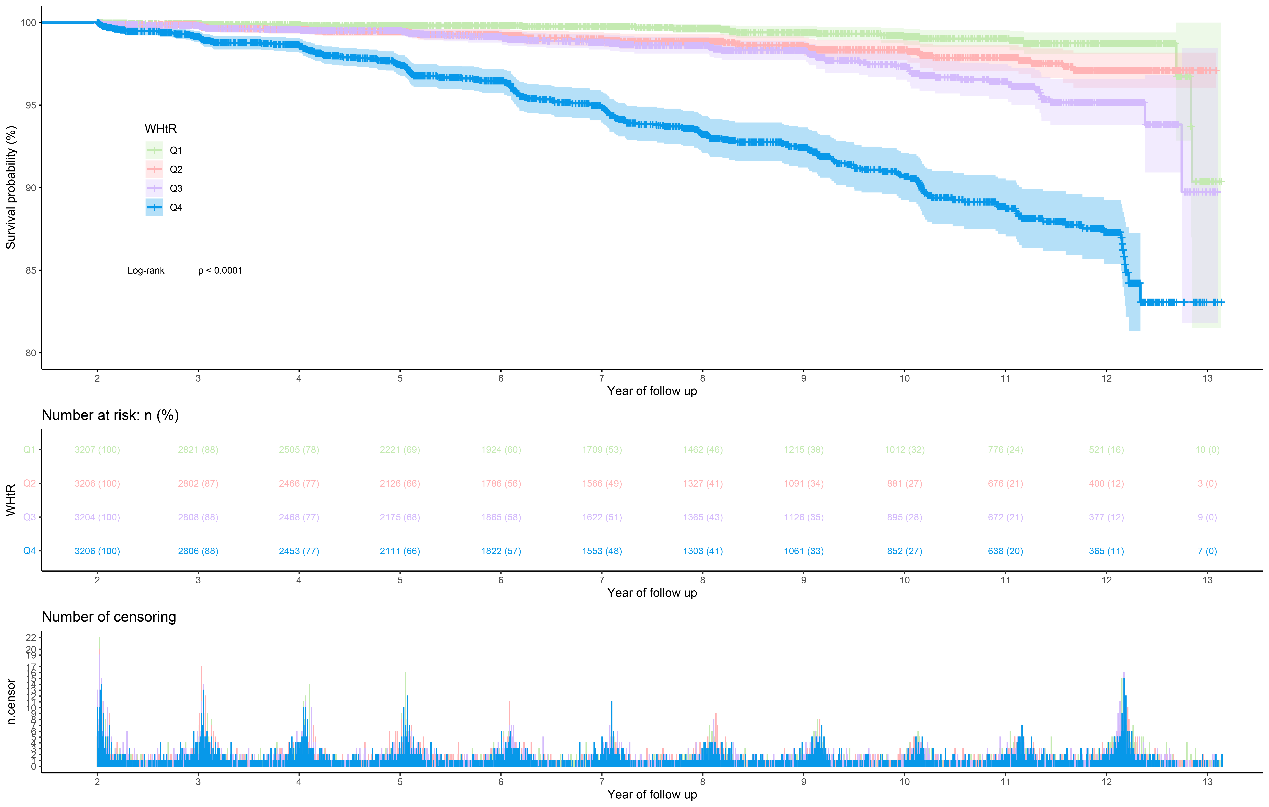


**Figure S3:** Kaplan-meier curve of WHtR quartiles over time. WHtR: waist-height ratio.
