# Supplementary material for: Assessing temporal differences of baseline body mass index, waist circumference, and waist-height ratio in predicting future diabetes
Source: Front Endocrinol (Lausanne). 2023 Jan 6;13:1020253. doi: 10.3389/fendo.2022.1020253 (PMC9852880; doi:10.3389/fendo.2022.1020253)
Supplement: Supplementary file 3 [file Table_1.docx]

Supplementary Table 1: Collinearity diagnostics steps of BMI with other covariates.

|  | VIF | | | |
| --- | --- | --- | --- | --- |
|  | **Step 1** | **Step 2** | **Step 3** | **Step 4** |
| BMI | 229.1 | 99.8 | 5 | 5 |
| Gender | 3.2 | 3.2 | 3.2 | 3.2 |
| Age | 1.4 | 1.4 | 1.4 | 1.4 |
| Height | 91 | 52.3 | 2.5 | 2.5 |
| Weight | 426.2 | 171 | NA | NA |
| WC | 1194.2 | NA | NA | NA |
| WHtR | 946.3 | 4.8 | 4.7 | 4.7 |
| ALT | 4.2 | 4.2 | 4.1 | 4.1 |
| AST | 3.3 | 3.3 | 3.3 | 3.3 |
| GGT | 1.5 | 1.5 | 1.5 | 1.5 |
| HDL-C | 1.8 | 1.8 | 1.8 | 1.8 |
| TC | 1.4 | 1.4 | 1.4 | 1.4 |
| TG | 1.8 | 1.8 | 1.8 | 1.8 |
| FPG | 1.5 | 1.5 | 1.5 | 1.5 |
| HbA1c | 1.3 | 1.3 | 1.3 | 1.3 |
| Fatty liver | 1.6 | 1.6 | 1.6 | 1.6 |
| Exercise habits | 1 | 1 | 1 | 1 |
| Drinking status | 1.3 | 1.3 | 1.3 | 1.3 |
| Smoking status | 1.4 | 1.4 | 1.4 | 1.4 |
| SBP | 5.6 | 5.6 | 5.6 | 1.4 |
| DBP | 5.7 | 5.7 | 5.7 | NA |

Abbreviations: Inf: infinity; VIF: Variance inflation factor; Other abbreviations as in Table ​1.

Note: VIF = 1/(1-R^2^).

Supplementary Table 2: Collinearity diagnostics steps of WC with other covariates.

|  | VIF | | | |  |
| --- | --- | --- | --- | --- | --- |
|  | **Step 1** | **Step 2** | **Step 3** | **Step 4** | **Step 5** |
| WC | 1194.2 | 6 | 6 | 6 | 2.1 |
| Gender | 3.2 | 3.2 | 3.2 | 3.2 | 3.2 |
| Age | 1.4 | 1.4 | 1.4 | 1.4 | 1.3 |
| Height | 91 | 52.6 | 2.8 | 2.8 | 2.5 |
| Weight | 426.2 | 170.1 | NA | NA | NA |
| BMI | 229.1 | 96.5 | 5.1 | 5 | NA |
| WHtR | 946.3 | NA | NA | NA | NA |
| ALT | 4.2 | 4.2 | 4.1 | 4.1 | 4.1 |
| AST | 3.3 | 3.3 | 3.3 | 3.3 | 3.3 |
| GGT | 1.5 | 1.5 | 1.5 | 1.5 | 1.5 |
| HDL-C | 1.8 | 1.8 | 1.8 | 1.8 | 1.8 |
| TC | 1.4 | 1.4 | 1.4 | 1.4 | 1.4 |
| TG | 1.8 | 1.8 | 1.8 | 1.8 | 1.8 |
| FPG | 1.5 | 1.5 | 1.5 | 1.5 | 1.5 |
| HbA1c | 1.3 | 1.3 | 1.3 | 1.3 | 1.3 |
| Fatty liver | 1.6 | 1.6 | 1.6 | 1.6 | 1.5 |
| Exercise habits | 1 | 1 | 1 | 1 | 1 |
| Drinking status | 1.3 | 1.3 | 1.3 | 1.3 | 1.3 |
| Smoking status | 1.4 | 1.4 | 1.4 | 1.4 | 1.4 |
| SBP | 5.6 | 5.6 | 5.6 | 1.4 | 1.4 |
| DBP | 5.7 | 5.7 | 5.7 | NA | NA |

Abbreviations: VIF: Variance inflation factor; Other abbreviations as in Table ​1.

Note: VIF = 1/(1-R^2^).

Supplementary Table 3: Collinearity diagnostics steps of WHtR with other covariates.

|  | VIF | | | |  |
| --- | --- | --- | --- | --- | --- |
|  | **Step 1** | **Step 2** | **Step 3** | **Step 4** | **Step 5** |
| WHtR | 946.3 | 4.8 | 4.7 | 4.7 | 1.7 |
| Gender | 3.2 | 3.2 | 3.2 | 3.2 | 3.2 |
| Age | 1.4 | 1.4 | 1.4 | 1.4 | 1.3 |
| Height | 91 | 52.3 | 2.5 | 2.5 | 2.4 |
| Weight | 426.2 | 171 | NA | NA | NA |
| WC | 1194.2 | NA | NA | NA | NA |
| BMI | 229.1 | 99.8 | 5 | 5 | NA |
| ALT | 4.2 | 4.2 | 4.1 | 4.1 | 4.1 |
| AST | 3.3 | 3.3 | 3.3 | 3.3 | 3.3 |
| GGT | 1.5 | 1.5 | 1.5 | 1.5 | 1.5 |
| HDL-C | 1.8 | 1.8 | 1.8 | 1.8 | 1.8 |
| TC | 1.4 | 1.4 | 1.4 | 1.4 | 1.4 |
| TG | 1.8 | 1.8 | 1.8 | 1.8 | 1.8 |
| FPG | 1.5 | 1.5 | 1.5 | 1.5 | 1.5 |
| HbA1c | 1.3 | 1.3 | 1.3 | 1.3 | 1.3 |
| Fatty liver | 1.6 | 1.6 | 1.6 | 1.6 | 1.5 |
| Exercise habits | 1 | 1 | 1 | 1 | 1 |
| Drinking status | 1.3 | 1.3 | 1.3 | 1.3 | 1.3 |
| Smoking status | 1.4 | 1.4 | 1.4 | 1.4 | 1.4 |
| SBP | 5.6 | 5.6 | 5.6 | 1.4 | 1.4 |
| DBP | 5.7 | 5.7 | 5.7 | NA | NA |

Abbreviations: VIF: Variance inflation factor; Other abbreviations as in Table ​1.

Note: VIF = 1/(1-R^2^).
